# Resistance Training Improves Hemodynamic Function, Collagen Deposition and Inflammatory Profiles: Experimental Model of Heart Failure

**DOI:** 10.1371/journal.pone.0110317

**Published:** 2014-10-23

**Authors:** Jadson P. Alves, Ramiro B. Nunes, Giuseppe P. Stefani, Pedro Dal Lago

**Affiliations:** 1 Laboratory of Physiology, Universidade Federal de Ciências da Saúde de Porto Alegre (UFCSPA), Porto Alegre, Rio Grande do Sul, Brazil; 2 Department of Physical Therapy, Universidade Federal de Ciências da Saúde de Porto Alegre (UFCSPA), Porto Alegre, Rio Grande do Sul, Brazil; Temple University, United States of America

## Abstract

The role of resistance training on collagen deposition, the inflammatory profile and muscle weakness in heart failure remains unclear. Therefore, this study evaluated the influence of a resistance training program on hemodynamic function, maximum strength gain, collagen deposition and inflammatory profile in chronic heart failure rats. Thirty-two male Wistar rats submitted to myocardial infarction by coronary artery ligation or sham surgery were assigned into four groups: sedentary sham (S-Sham, n = 8); trained sham (T-Sham, n = 8); sedentary chronic heart failure (S-CHF, n = 8) and trained chronic heart failure (T-CHF, n = 8). The maximum strength capacity was evaluated by the one maximum repetition test. Trained groups were submitted to an 8-week resistance training program (4 days/week, 4 sets of 10–12 repetitions/session, at 65% to 75% of one maximum repetition). After 8 weeks of the resistance training program, the T-CHF group showed lower left ventricular end diastolic pressure (*P*<0.001), higher left ventricular systolic pressure (*P*<0.05), higher systolic blood pressure (*P*<0.05), an improvement in the maximal positive derivative of ventricular pressure (*P*<0.05) and maximal negative derivative of ventricular pressure (*P*<0.05) when compared to the S-CHF group; no differences were observed when compared to Sham groups. In addition, resistance training was able to reduce myocardial hypertrophy (*P*<0.05), left ventricular total collagen volume fraction (*P*<0.01), IL-6 (*P*<0.05), and TNF-α/IL-10 ratio (*P*<0.05), as well as increasing IL-10 (*P*<0.05) in chronic heart failure rats when compared to the S-CHF group. Eight weeks of resistance training promotes an improvement of cardiac function, strength gain, collagen deposition and inflammatory profile in chronic heart failure rats.

## Introduction

Chronic heart failure (CHF) is a complex clinical syndrome produced by structural and functional disorders of the heart [1]. Patients with CHF present exercise intolerance; this limitation cannot be solely attributed to cardiac and pulmonary damage [2]. Additionally, abnormalities in skeletal muscle probably contribute to the development of symptoms and exercise intolerance in CHF [3]–[5].

Damage to the cardiac muscle and to the extracellular matrix leads to changes in the size, shape, and function of the left ventricle, resulting in changes in the entire heart, in a process named cardiac remodeling [6], which affects prognosis and survival in patients with CHF [7]. The increase in fibrous tissue has been observed in animal models of heart failure, and there is also evidence that the same mechanism occurs in human heart failure [8]. Additionally, the extent of damage in collagen is correlated with the degree of expansion of infarction [9]. The accumulation of collagen in places remote to myocardial infarction (MI) is the main negative component of structural remodeling in heart failure of an ischemic origin [10].

The positive effects of exercise on the pathophysiology of CHF have been reported in recent years [11], [12], and exercise protocols, such as resistance training (RT), appear to be an auxiliary strategy combined with aerobic training in cardiac rehabilitation programs [13]. The regular practice of physical exercise not only reverses the musculoskeletal changes attributed to physical inactivity, but also inhibits the inflammatory process induced by CHF by increasing anti-inflammatory agents [14].

In response to the effects caused by a physical training program, the heart will develop adaptations of the myocardium, causing a physiological state of cardiac remodeling. These morphological changes may differ depending on the type of training and are clinically characterized by changes in heart size and shape, due to increased load [15]. The RT prescribed with appropriate intensity is associated with a lower hemodynamic load than most of the prescriptions related to submaximal aerobic training, and still may have an additional benefit in reducing the peripheral limitations, which is a common feature found in patients with CHF [16].

Some studies have demonstrated the benefits of RT in patients with CHF [17]–[20], but the effects of RT on cardiac remodeling and inflammatory profiles are poorly understood. In animal models of CHF, to the best of our knowledge, there are no studies that have evaluated the physiological responses of RT. Therefore, we hypothesized that RT and, consequently, the strength gain could be associated with improvements of cardiac function in rats with CHF. Consequently, the aim of the present study was to evaluate the effects of RT on hemodynamic function, maximal strength gain, collagen deposition and inflammatory profile in rats with CHF.

## Methods

### Ethical Approval

The investigation followed the ethical rules established by the Guide for the Care and Use of Experimental Animals published by the National Institute of Health (NIH publication no. 85-23, revised in 1996). All of the procedures outlined in this study were approved by the Ethics Committee Research of the UFCSPA (protocol 007-10).Thirty-two male Wistar rats (220 to 270 g; 90 days of age) obtained from the Animal Breeding Unit of the Universidade Federal de Ciências da Saúde de Porto Alegre were housed under standard conditions (food and water *ad libitum*, 12∶12-h light-dark cycle; 22°C).

### Surgery to induce MI

Rats were anesthetized with xylazine (12 mg/kg i.p.) and ketamine (90 mg/kg i.p.) and artificially ventilated (SamWay VR 15; 60 breaths/min). After thoracotomy, coronary artery ligation (CAL) was performed to induce MI. The sham animals underwent the same procedure without artery ligation, as described previously [21].

### Experimental design

After MI, the rats were allowed a minimum of 6 weeks of recovery (the necessary time to achieve the development of the CHF state) [22], [23]. The animals were assigned to four experimental groups: sedentary sham (S-Sham, n = 8), trained sham (T-Sham, n = 8), sedentary CHF (S-CHF, n = 8), or trained CHF (T-CHF, n = 8).

### Resistance training program

**Figure 1:**
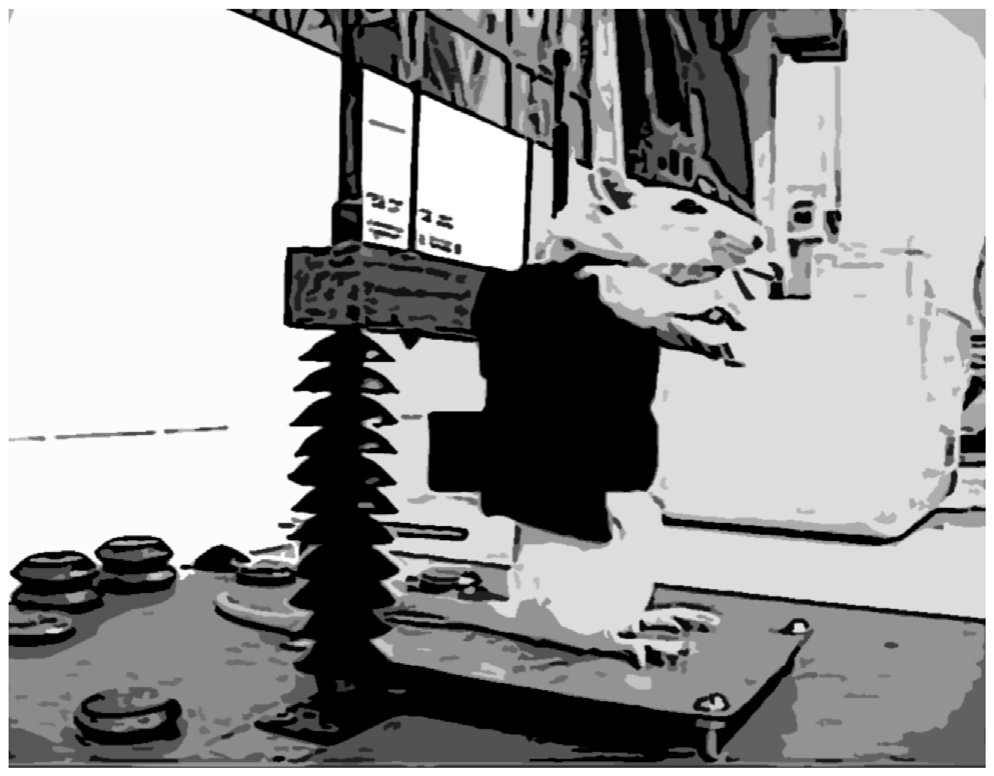
Representative illustration of the apparatus used for resistance training.

Training groups underwent a 1 week familiarization period (1 set of 10 repetitions, 3 days/week) in the adapted apparatus for RT (Figure 1) [24]. The rats were placed in a neoprene vest leaving it in an upright position on their lower limbs. An electrical stimulus (4–5 mA, 1 second duration, with a 3 second interval between each repetition) was applied to the rat's tail through a surface electrode. As a result, the animals extended their legs repeatedly, which lifted the weight on the exercise apparatus. To determine the training workload, all rats were submitted to a one repetition maximum test (1RM); the 1RM was determined as the maximum weight lifted with the exercise apparatus. The RT program was based on 4 sets of 10–12 repetitions, 65 to 75% of the 1RM, a rest period of 90 s between sets, 4 times per week, for a total of 8 weeks. After the 1RM test, the animals were exercised at 65% of the 1RM in the first week and the workload was increased in the following weeks to 75% of the 1RM. The 1RM test was performed every 2 weeks. The intensities of this protocol are classified as moderate and high intensity for the percentage of 65 to 75% of 1RM according to the guidelines of the American College of Sports Medicine [25].

### Surgical preparation for hemodynamic evaluation

Forty-eight hours 48 h after the last exercise session, the animals were anesthetized with xylazine (12 mg/kg i.p.) and ketamine (90 mg/kg i.p.), and a polyethylene catheter (PE-50) was placed into the right carotid artery. The arterial pressure was recorded and the catheter was positioned into the left ventricle to perform the ventricular pressure record. The data were registered by a pressure transducer (strain-gauge, Narco Biosystem Miniature Pulse Transducer RP-155, Houston, Texas, USA), coupled to a pressure amplifier (Stoelting, wood dated, Illinois, USA). Pressure analogical signals were digitalised by a data-acquisition system (CODAS-Data Acquisition System, Akron, OH, USA) with a sampling rate of 2,000 Hz. These data were used to determine diastolic blood pressure (DBP), systolic blood pressure (SBP), mean blood pressure (MBP), heart rate (HR), left ventricular systolic pressure (LVSP), left ventricular end-diastolic pressure (LVEDP), and left ventricular maximum positive and negative dP/dt (+dP/dt_max_, −dP/dt_max_), as previously described [14].

### Blood samples and muscle collection

Blood samples were drawn from the catheter positioned in the right carotid artery, collected into a 1.5 ml tube containing 3.2% sodium citrate (1∶9 vol/vol), centrifuged at 500×*g* for 10 min at 4°C and the plasma was stored at −22°C. Animals were killed after blood drawing through an intravenous infusion of an overdose of the anesthetic pentobarbital (80 mg/kg, i.p.) [26].

### Infarct size, cardiac hypertrophy and pulmonary and hepatic congestion

Hearts were removed and weighed, without blood within the chamber and without atria. The size of the infarct was determined by the planimetry [27]. To evaluate cardiac hypertrophy, organ weights were expressed as a proportion of body weight (tissue weight/body weight – mg/g). To determine pulmonary and hepatic congestion, the lungs and liver of each animal were removed, weighed, and dehydrated (80°C) for 48 h, and then weighed again to evaluate the water percentage, using the following formula:
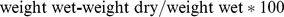



### Analysis of collagen content

The hearts were sectioned in a cryostat (6 µm) and were stained with collagen-specific picrosirius red (PSR) for measurements of the content of interstitial collagen [28]. The images were obtained using a polarized light microscope [29] (Olympus BX51) with a camera attached (Olympus DP72, 40X objective). For image acquisition and quantification, the image pro plus software version 4.5 (Media Cybernetics, Silver Spring, Maryland, USA) was used. For each animal, 40 fields were selected, with the chosen fields being located far from the infarcted area and from the pericardial region, in order to determine the percentage of collagen volume fraction (CVF). The collagen volume fraction was defined as the sum of all stained interstitial collagen tissue areas divided by the entire tissue area [30].

### Determination of plasma levels of cytokines

TNF-α, IL-6 and IL-10 plasma levels were determined by a multiplex bead array using Milliplex MAP rat cytokine kits (RCYTO-80K, Millipore - Billerica, MA, USA). Milliplex MAP is based on the Luminex xMAP technology as recommended by the manufacturers. All cytokines are reported as pg/ml.

### Statistical analysis

All data are expressed as mean ± SE. The Shapiro–Wilk's normality test was performed. Data were compared among groups by two-way ANOVA followed by the Student-Newman-Keuls post hoc test, and Pearson correlation was also used. A P value of <0.05 was considered significant. The GraphPad Prism 5.03 program (GraphPad Software, San Diego, CA) and Sigma Plot 12.0 (Systat Software Inc., San Jose, USA) for Windows were used as a computational tool for data analysis.

## Results

### Total mortality

Mortality in MI-induced CHF rats, during or immediately after surgery, was 35%. There were no deaths or behaviors associated with stress or adverse effects in rats that participated in the RT program in both the sham and CHF groups.

### Maximal strength

**Figure 2:**
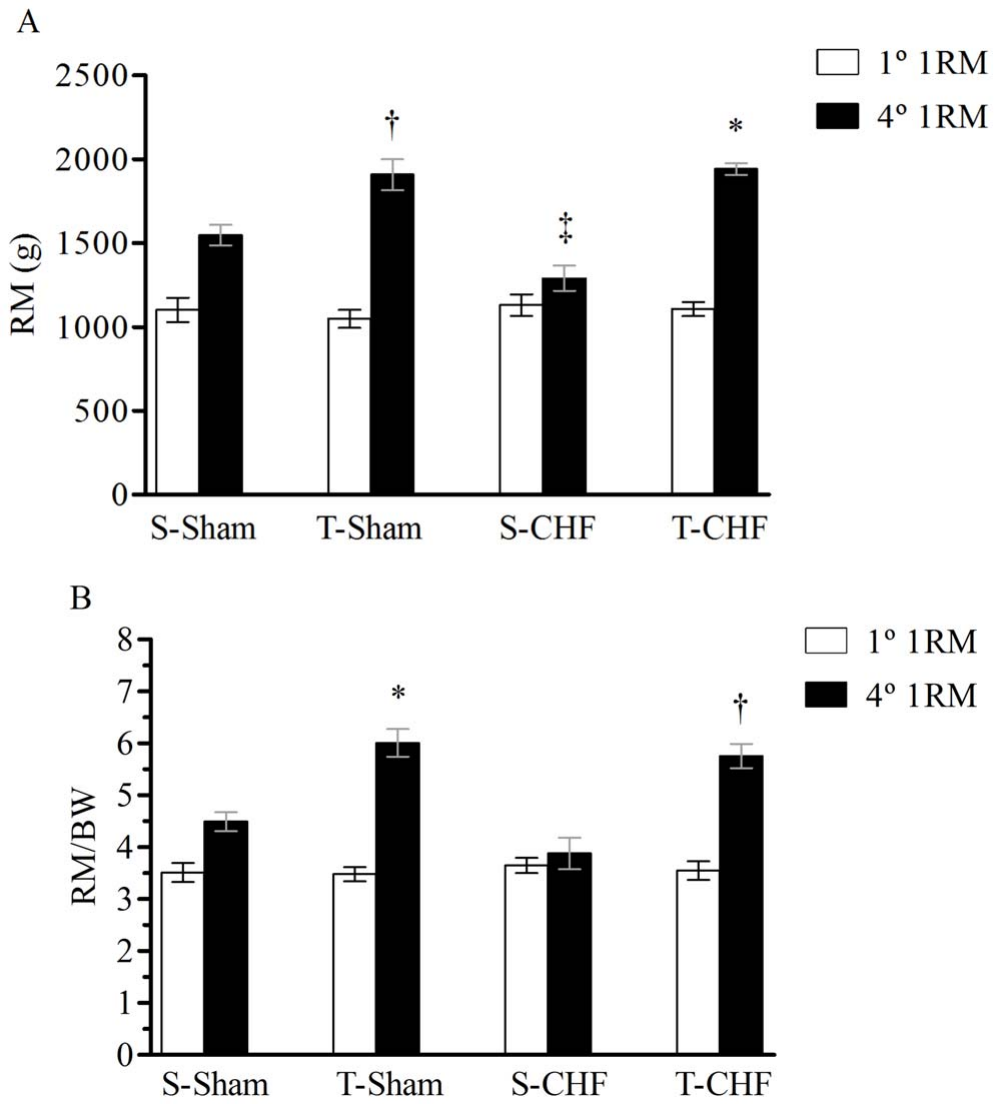
Absolute values for one repetition maximal test (1RM). (**A**). Relative values for one repetition maximal test (1RM) (**B**). Values are means ± SE; n = 8 for all groups. S-Sham, sedentary sham rats; T-Sham, training sham rats; S-CHF, sedentary chronic heart failure rats; T-CHF, training chronic heart failure rats. *P<0.001 compared to sedentary groups. †*P*<0.01 compared to sedentary groups ‡*P*<0.05 compared to S-Sham group.

Figure 2A shows the RM test and a progressive increase in the absolute weight lifted by the trained groups compared to the control groups. All groups had similar values for 1RM at the beginning of the protocol. In the last test of 1RM (the fourth 1RM), the trained groups had an increase in strength when compared to sedentary groups. The S-Sham group had a moderate increase in absolute load when compared to S-CHF, but this difference was not significant when the load was normalized by body weight 1RM/BW (g lifted/g BW; Figure 2B), showing that only the trained groups had a significant increase in the load lifted in the last 1RM test relative to body weight.

### Body weight, infarct size, heart hypertrophy, pulmonary and hepatic congestion

All of these data are summarized in Table 1. The initial body weights and final body weights were similar among the four groups in both the pre- and post-training period. There were no differences in the infarcted area between the CHF groups. Pulmonary congestion (PC) was higher in the S-CHF group when compared to Sham groups; however, the T-CHF group showed lower pulmonary congestion when compared to the S-CHF group. Also, there were no differences between the sham groups and the T-CHF group in this variable.

**Table 1:** Body weight, morphometric cardiac characteristics, infarct area and pulmonary and hepatic congestion of sham-operated rats and rats with left ventricular dysfunction.

Variables	S-Sham	T-Sham	S-CHF	T-CHF
Initial Body Weight, g	253.4±3	250.0±4	253.0±4	258.0±4
Final Body Weight, g	354.0±10	321.0±5	345.0±13	340.0±9
Infarcted Area %	----------	----------	41.5±2	39.3±1
MW/BW mg/g	3.1±0.05^†^	3.5±0.10	4.2±0.16*	3.8±0.09
LVW/BW mg/g	2.4±0.05^†^	2.8±0.09	3.2±0.10^‡^	3.1±0.06
RVW/BW mg/g	0.69±0.03	0.64±0.04	1.02±0.09*	0.74±0.05
Pulmonary Congestion (PC) %	73.2±0.57	70.7±1.2	77.0±1.3*	71.9±1.1
Hepatic Congestion (HC) %	70.8±0.24	70.3±0.19	70.8±0.38	70.2±0.26

Values are means ± SE; n = 8 for all groups. S-Sham, sedentary sham rats; T-Sham, training sham rats; S-CHF, sedentary chronic heart failure rats; T-CHF, training chronic heart failure rats. MW/BW, myocardial weight-to-body weight ratio; LVW/BW, left ventricle-to-body weight ratio and RVW/BW, right ventricle-to-body weight ratio. *P<0.05 compared to all groups. ^†^ *P*<0.05 compared to trained groups. ^‡^ *P*<0.05 compared to Sham groups.

The S-CHF group showed higher cardiac hypertrophy, expressed by the increased weight of the myocardium (MW) normalized by body weight (MW/BW), compared to the sham groups. In addition, the T-CHF group had lower cardiac hypertrophy when compared to S-CHF, which demonstrated that the RT was able to attenuate the pathological hypertrophy. Furthermore, no differences were observed in cardiac hypertrophy between the T-CHF and T-Sham groups. Other comparisons are shown in Table 1.

The left ventricular hypertrophy, expressed by the increased weight of the left ventricle (LV) normalized by body weight (LVW/BW), was higher in the S-CHF group when compared to the sham groups. However, there were no differences between the T-CHF group and the S-CHF group. No difference was observed in LV hypertrophy between the T-Sham and T-CHF groups. More results are listed in Table 1.

Right ventricle hypertrophy (RVW/BW) was higher in the S-CHF group compared to the sham groups. The T-CHF group showed lower RV hypertrophy when compared to the S-CHF group, suggesting an attenuation of pathological hypertrophy due to training. Moreover, the T-CHF group showed no difference in hypertrophy in RV compared to the sham group.

### Hemodynamic parameters

All of these data are summarized in Table 2. The S-CHF group presented values of LVEDP above 20 mmHg, characterizing the presence of significant ventricular dysfunction compared to the sham groups. However, as shown in Table 2, the LVEDP was lower in the T-CHF group when compared to the S-CHF group. No difference was found in the LVEDP in the T-CHF group when compared to the sham groups.

**Table 2:** Mean blood pressure, diastolic blood pressure, systolic blood pressure, left ventricular end-diastolic pressure, ventricular systolic pressure, left ventricular maximum change in pressure over time and left ventricular minimum change in pressure over time of sham-operated rats and rats with left ventricular dysfunction.

Variables	S-Sham	T-Sham	S-CHF	T-CHF
MBP (mmHg)	99.3±3.9	101.8±4.5	85.7±3.2	99.2±5.8
DBP (mmHg)	85.9±3.8	89.0±4.4	76.4±3.2	88.0±5.5
SBP (mmHg)	112.0±4.3	112.09±4.7	94.19±3.3*	108.08±6.1
LVEDP (mmHg)	5.52±0.65	4.50±0.89	24.20±1.5*	7.62±1.4
LVSP (mmHg)	113.00±6	107.80±4	88.14±3*	102.61±6
+ dP/dtmax (mmHg)	6071±603	5926±750	3548±320*	5290±599
− dP/dtmax (mmHg)	4154±376	4024±358	2495±159*	3519±414

Values are means ± SE; n = 8 for all groups. S-Sham, sedentary sham rats; T-Sham, training sham rats; S-CHF, sedentary chronic heart failure rats; T-CHF, training chronic heart failure rats. Mean blood pressure (MBP), diastolic blood pressure (DBP), systolic blood pressure (SBP), left ventricular end-diastolic pressure (LVEDP) left ventricular systolic pressure (LVSP), maximal positive derivative of ventricular pressure (+dP/dt_max_) and maximal negative derivative of ventricular pressure (−dP/dt_max_). *P<0.05 compared to all groups.

The LVSP was lower in the S-CHF group when compared to all groups (Table 2). The T-CHF group showed an improvement in +dP/dt_max_ and −dP/dt_max_ when compared to the S-CHF group. The SBP was lower in the S-CHF group when compared to all groups.

### Myocardial collagen

**Figure 3:**
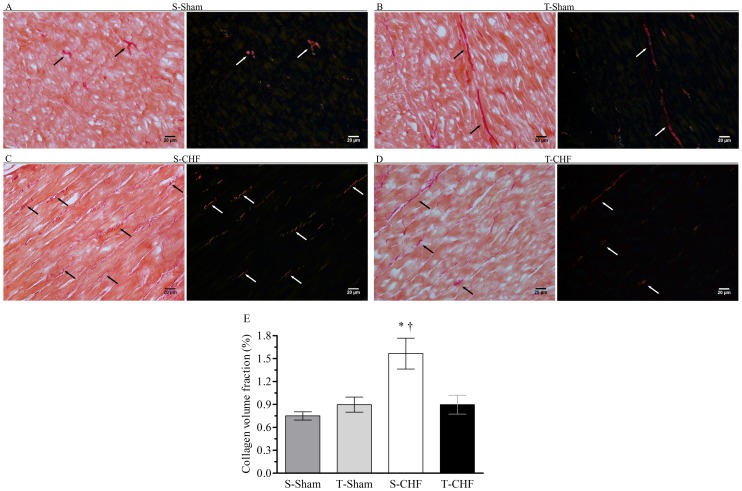
Representative images of ventricular sections stained with Picrosirius (A–D) and statistical analysis (E). Values are means ± SE. The arrows indicate collagen detected in polarized light. **A**. S-Sham, sedentary sham rats (n = 7); **B**. T-Sham, training sham rats (n = 8); **C**. S-CHF, sedentary chronic heart failure rats (n = 8); **D**. T-CHF, training chronic heart failure rats (n = 8). *P<0.01 compared to trained groups. †*P*<0.001 compared with S-Sham group.

Figure 3 shows the sections stained with PSR non-infarcted LV under bright field and polarized light for the S-Sham, T-Sham, S-CHF and T-CHF groups, as well as for the total CVF. Total CVF was higher in the S-CHF group compared to S-Sham, T-Sham and T-CHF groups. Furthermore, the T-CHF group showed no difference in total CVF when compared to the sham groups.

### Inflammatory Profile

**Figure 4:**
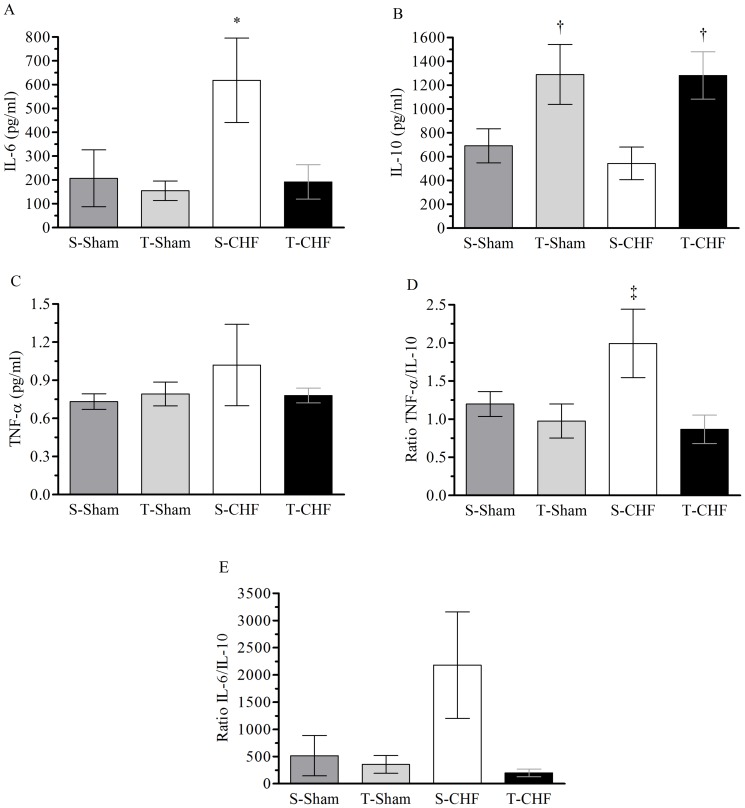
Plasma levels of IL-6, IL-10, TNF-α, TNF- α to IL-10 ratio and IL-6 to IL-10 ratio (A–E). Values are means ± SE. Figure A) S-Sham, sedentary sham rats (n = 5); T-Sham, training sham rats (n = 5); S-CHF, sedentary chronic heart failure rats (n = 5); T-CHF, training chronic heart failure rats (n = 6). Figure B, C and D, n = 6 for all groups. Figure E, n = 5 for all groups. *P<0.05 compared to all groups. †*P*<0.05 compared to sedentary groups. ‡*P*<0.05 compared to trained groups.

Figure 4 shows the plasma concentrations of IL-6, IL-10 and TNF-α, as well as the TNF-α to IL-10 and IL-6 to IL-10 ratios. IL-6 was higher in the S-CHF group in comparison with all other groups. In contrast, plasma levels of IL-10 were higher in the T-CHF group when compared to the S-CHF group. We observed a decrease in TNF-α/IL-10 in the T-CHF group when compared with the S-CHF group. In addition, no differences were observed in the TNF-α/IL-10 ratio between the T-CHF group and the Sham groups. The IL-6/IL-10 and plasma levels of TNF-α had no difference among groups. Cytokine analyses were performed only with plasma samples feasible.

## Discussion

The main findings of the present study were the improvement of maximal strength gain, hemodynamic function, collagen deposition and the inflammatory profile in rats with CHF subsequent to MI who were submitted to the 8 week RT protocol. We demonstrate that the RT protocol in CHF rats was able to improve cardiac function, as demonstrated by the increased LVSP and SBP, the improved +dP/dtmax and -dP/dtmax, and the decrease in LVEDP. The RT protocol was able to attenuate cardiac hypertrophy, left ventricular collagen volume fraction and reduced IL-6 and the ratio of TNF-α/IL-10; also, there was increased IL-10 in CHF rats. The results presented here are very consistent and only related to the HF animal model; the improvement in cardiac function observed here may not occur in patients with HF. In this context, in the CHF animal model, the injury is mainly central rather than peripheral, which is different in humans with HF syndrome. In fact, this could be, at least in part, one of the reasons for the results presented here, which demonstrate higher improvements in cardiac function after RT in the animal model [31]. On the other hand, the load during RT in cardiac rehabilitation programs is seated in lower levels (40-60%, 1RM) than that used here (75%, 1RM).

The maximal strength gain in CHF trained rats was an important result found in this study. It is well known that muscle weakness is an independent risk factor for mortality in patients with CHF; therefore, the increased strength becomes a survival predictor in this population suffering from the harmful scenario imposed by CHF [32], [33]. In addition, other studies have shown that the increase in muscular strength promotes improvement in clinical status [17], and improvement in functional and physiological capacity to perform activities of daily living [20].

The left coronary artery ligature produces marked left ventricular dysfunction that is directly related to the size of the infarcted area [21], [22], [34] and simulates the most common cause of CHF, which can negatively alter hemodynamic function. In this model, MI areas greater than 30% represent severe myocardial injury, which results in sustained hemodynamic dysfunction with a higher LVEDP, lower LVSP and heart hypertrophy [14], [26]. In the present study, the infarcted area was around 40%, which is associated with severe impairment of the of left ventricular function, producing a severely ill state of the left ventricle, with increases in the end-diastolic pressure higher than 20 mmHg, characterizing the development of severe CHF [35].

In a previous study developed in our laboratory [14], a reduction of 12% in LVEDP was demonstrated in rats with CHF that were submitted to swimming protocol training. More recently, a reduction of 33% in LVEDP and an increase in LVSP of 21% was demonstrated in rats with CHF submitted to respiratory muscle training [26]. These results are similar to the responses found in the present study; therefore, we observed that the RT protocol in rats with CHF was able to reduce 68% in LVEDP and increase 14% in LVSP. Moreover, Pinter *et al*. [36] reported that healthy animals submitted to the RT protocol with intensity of 75% of 1RM test showed an improvement in cardiac performance, probably due to better myocardial perfusion as a result of the increase in systolic pressure of the heart, which increases the dP/dt values during the RT protocol. These results support, in part, the improved cardiac performance found in animals with CHF who underwent the RT protocol in our study. In addition, positive effects of RT on ventricular function, cardiac hypertrophy and maximal strength gain in rats with no left ventricular dysfunction or other cardiovascular disease have been reported [37].

It was also found that RT was able to improve +dP/dtmax and -dP/dtmax (33% and 29%, respectively) in CHF rats. These findings add to those observed in clinical reports, which have clearly demonstrated that RT does not cause a reduction of LV contractility function or enhance myocardial deterioration in the patients with CHF [18], [19]. The improvement in cardiac function could be a result of hemodynamic parameters ameliorating the T-CHF group. These results are in agreement with clinical studies which demonstrated that RT can lead to a positive response in muscle strength and endurance, without the occurrence of adverse effects on hemodynamics [16], [38].

Reduction of the right-ventricle compensatory hypertrophy was found after training (28%) in CHF rats; this is associated with decreased pulmonary congestion (*r* = 0.41; *P* = 0.01). These results demonstrate that pulmonary congestion is negatively associated with the RV hypertrophy. Interestingly, Musch *et al*. [35] reported that rats with severe left ventricular dysfunction, characterized by increasing LVEDP, showed significant pulmonary congestion. In the present study, we observed that the CHF rats undergoing the RT protocol showed reduced pulmonary congestion together with attenuated LVEDP.

The changes in interstitial collagen after MI have gained attention in recent years [8], [10], [39], but the effects of exercise on changes in interstitial collagen after MI are rarely observed in the literature [28], [40], and models of RT for small animals with CHF have not existed until now. Perhaps, higher myocardial collagen content together with the increases in LVEDP are associated with pathologic hypertrophy.

The accumulation of interstitial collagen in rats with CHF after the moderate and high intensity RT protocol (65–75% of 1RM) was able to attenuate the CVF total in comparison to the sedentary group with CHF, suggesting that the reverse of cardiac remodeling occurs. These data corroborate the findings of Xu *et al*. [28], which demonstrated that, after MI, animals submitted to physical exercise had lower CVF totals when compared to sedentary post-MI animals. Our results also corroborate the study of Emter & Baines [40], which demonstrated that physical exercise can prevent pathological remodeling and dysfunctional mitochondria in HF, through attenuation of the accumulation of collagen and the maintenance of a positive inotropic state in pigs with HF.

The increase in ventricular filling pressure has been associated with impaired ventricular systolic function and reduced ventricular compliance by collagen accumulation and fibrosis [41]. In our study, the T-CHF group showed an improvement in ventricular function compared to the S-CHF group, observed by the reduced LVEDP, as previously mentioned, demonstrating that the attenuation of collagen accumulation in animals with CHF that underwent RT may influence the improvement of this parameter. Similar results were observed in the study of Yengo *et al*. [42], who showed that after 10 weeks of training on the treadmill, exercise can minimize the deleterious effects of fibrosis seen post-MI, reducing the stiffness characteristics of the right ventricle that can be associated with abnormalities of diastolic function after heart attack.

The benefits of exercise on inflammatory markers are primarily based on responses to aerobic training, and, to the best of our knowledge, studies demonstrating the inflammatory responses of the isolated practice of RT in CHF have not been performed. Elevated levels of pro-inflammatory cytokines such as TNF-α and IL-6 have been associated with an increased disease severity and poor prognosis in patients with CHF [43]. Interestingly, in our study, we observed that the inflammatory profile in rats with CHF that underwent moderate to high intensity RT protocol (65–75% of 1RM) was able to attenuate the plasma levels of IL-6 when compared with the sedentary CHF group and differences were not observed in the plasma levels of TNF-α between the groups. These results support, in part, the findings of other studies that have shown that aerobic exercise is able to reduce levels of the pro-inflammatory cytokine IL-6 [44], [45].

In addition, it was also observed that the RT was able to promote an anti-inflammatory effect in animals with CHF by increasing the plasma levels of IL-10, resembling those observed in aerobic training [14]. Furthermore, in the present study, the TNF-α/IL-10 ratio was lower in the T-CHF group when compared to S-CHF, demonstrating that the RT has a positive effect on the inflammatory process imposed by CHF. Similar responses were found in the study of Batista *et al*. [46], which showed an increase in the IL-10/TNF-α ratio in the skeletal muscle of CHF rats submitted to aerobic training, demonstrating the importance of exercise as an anti-inflammatory strategy. In addition, patients with acute myocardial infarction showed higher TNF-α/IL-10 ratios when compared to the control group, demonstrating an important inflammatory imbalance [47]. Thus, physical exercise can be considered an anti-inflammatory strategy to ameliorate the balance of this immunological complex system.

The limitation of this study was the lack of histological assessment in skeletal muscle. This parameter would help to achieve a better understanding of the effect of RT on skeletal muscle fibers, in both trained and untrained CHF rats. Another limitation was the lack of expression levels of IL-6, IL-10 and TNF-α in the hearts of healthy animals and in those with CHF, which may explain the changes in hemodynamic parameters of the T-CHF group. Also, some animals with a large infarcted area did not present ventricular dysfunction; however, as we can see from the results on LVEDP and LVSP, both were reduced, which probably represents ventricular dysfunction.

In conclusion, a regular program of 8 weeks of RT in Wistar rats with CHF induced by MI was able to improve the hemodynamic function, reduce pulmonary congestion, increase maximum strength gain, attenuate cardiac hypertrophy, and improve collagen deposition in the LV and inflammatory profiles. These results demonstrate an important contribution of the RT in an experimental model of CHF. We suggest that RT may be an effective therapeutic strategy for a cardiac rehabilitation program. Further studies are needed to understand the other possible benefits of RT in CHF through researches that analyze cellular and molecular mechanism-related adaptations of cardiac muscle, skeletal muscle and blood vessels after RT alone or in combination with endurance training.
